# Traditional Chinese medicine for the treatment of pulmonary fibrosis: A protocol for systematic review and meta-analysis of overview

**DOI:** 10.1097/MD.0000000000021310

**Published:** 2020-07-31

**Authors:** Li-Juan Li, Xuan Chen, Wen-Na Yang, Xiang-Mei Xu, Li-Ying Lu, Jie Wang, Yi-Xuan Kong, Jing-Hui Zheng

**Affiliations:** a Graduate School, Guangxi University of Chinese Medicine; b Department of Geriatrics, Ruikang Affiliated Hospital of Guangxi University of Chinese Medicine, Nanning, Guangxi, China.

**Keywords:** curative effect, re-evaluation of systematic evaluation, Traditional Chinese Medicine, pulmonary fibrosis

## Abstract

**Background:**

Since December 2019, there have been many cases of viral pneumonia of unknown causes in Wuhan City, Hubei Province. During the period of novel coronavirus, according to the observation of limited autopsy and biopsy pathological results, pulmonary interstitial fibrosis appeared in some pathological changes of lung. Idiopathic pulmonary fibrosis (IPF) is a chronic progressive interstitial pneumonia with unknown etiology and pathological changes limited to the lung. At present, there is still a lack of reevaluation of systematic evaluation of traditional Chinese medicine treatment IPF. Therefore, a systematic re-evaluation of the systematic evaluation of traditional Chinese medicine in the treatment of pulmonary fibrosis may help to understand the effective treatment scheme of traditional Chinese medicine in the treatment of pulmonary fibrosis and provide more reliable evidence for the first-line clinicians to treat novel coronavirus.

**Methods:**

We will search 3 foreign electronic databases (Cochrane Library, Embase, PubMed) and 4 Chinese electronic databases (China National Knowledge Infrastructure [CNKI], WangFang Database, Chinese Biomedical Literature Database [CBM], and Chinese Scientific Journal Database [VIP]) to collect potential systematic reviews from their inceptions to February 2020. The language of publication is limited to Chinese or English. We will consider SRs and meta-analysis of Traditional Chinese Medicine for the Treatment of pulmonary fibrosis. Two reviewers will identify relevant studies, and then assess the methodological quality by assessment of multiple systematic reviews-2 tool. Using the Preferred Reporting Items for Systematic Reviews and Meta-Analyses (PRISMA) report checklist to assess the quality of reports included in the study. In order to better evaluate the systematic evaluation included in this research, risk of bias in systematic review tool is included in this research to evaluate the methodological quality. The quality of evidence of the included systematic reviews was assessed by the Grading of Recommendations Assessment, Development and Evaluation (GRADE) approach. The Primary outcomes include: Clinical total effective rate, curative effect of TCM symptoms, pulmonary function and blood gas analysis.

**Results:**

The results of this study will be published in a peer-reviewed journal.

**Conclusions:**

We expect to obtain reliable evidence from systematic analysis of traditional Chinese medicine treatment of pulmonary fibrosis in an available and useful document.

**Registration number:**

INPLASY202060029

## 1 Introduction

Since December 2019, there have been many cases of viral pneumonia of unknown causes in Wuhan City, Hubei Province.^[1]^ On February 11, the World Health Organization (WHO) officially named the disease caused by novel coronavirus infection as “COVID-19”. The new coronavirus and SARSr-CoV and MERSr-CoV belong to the coronavirus. Fever, dry cough and fatigue are the main manifestations of the cases. Severe patients may develop dyspnea, acute respiratory distress syndrome, etc.^[2]^ During the period of novel coronavirus, according to the observation of limited autopsy and biopsy pathological results, pulmonary interstitial fibrosis appeared in some pathological changes of lung.^[3]^

Idiopathic pulmonary fibrosis (IPF) is a chronic progressive interstitial pneumonia with unknown etiology and pathological changes limited to the lung. Its Histopathology or high-resolution CT manifestations are common interstitial pneumonia, with dry cough without sputum or only a small amount of foam sputum, chronic progressive dyspnea and systemic fatigue as the main clinical symptoms.^[4]^ IPF mainly occurs in adults, males outnumber females, and most of them have a history of smoking.^[5]^ Moreover, IPF is related to family gene abnormality and is a malignant progressive interstitial pneumonia.^[6]^ The typical manifestation is occult dyspnea in patients aged 60 to 70 years old.^[7]^ Pulmonary fibrosis is the replacement of healthy tissues by altered extracellular matrix, destruction of alveolar structure, resulting in decreased lung compliance, interruption of gas exchange, and eventually respiratory failure and death.^[8]^ The average survival rate of clinical manifestations is 2 to 5 years.^[9]^ There is no name for pulmonary fibrosis in ancient Chinese medical literature. According to Chinese medical literature and clinical manifestations of the disease, doctors attribute it to diseases such as “lung flaccidity”, “lung arthralgia”, “asthma”, “cough” and “lung distension”, while most doctors attribute the disease to “lung flaccidity.”^[10]^ Severe acute respiratory syndrome (SARS), which broke out as early as 2002, can lead to pulmonary fibrosis due to the further aggravation of pulmonary exudation.^[11]^ Studies have shown that SARS may cause the proliferation of fibrous cells in alveolar septa to form septa fibrosis, which continuously fuse with each other, further compress alveolus to shrink them, eventually turning large areas of lung tissue into solid Fibrosis areas, completely losing ventilation/ventilation function, and causing extremely difficult breathing.^[12,13]^ The results of Zhang and Zhang^[14]^ clinical trials show that the traditional Chinese medicine compound 861 granule is effective in treating pulmonary fibrosis or pulmonary fibrosis caused by SARS. The results of He et al^[15]^ clinical trial show that the combination of traditional Chinese and western medicine can improve the patient's condition and shorten the course of disease. Chest imaging shows that the degree of pulmonary fibrosis is reduced. The treatment of SARS by integrated traditional Chinese and western medicine is generally superior to the treatment of western medicine alone in terms of fever, improvement of dyspnea and other symptoms, acceleration of lung lesion absorption, reduction of complications and so on.^[16]^

After first-line clinicians took drugs, it was found that traditional Chinese medicine had obvious effect on novel coronavirus. Therefore, the experts on traditional Chinese medicine medical treatment and rehabilitation in state administration of traditional Chinese medicine recommended comprehensive intervention strategies based on traditional Chinese medicine, traditional non-drug therapy, psychology, diet and exercise techniques after systematic demonstration.^[17]^ In recent years, with the development of traditional Chinese medicine research, a large number of traditional Chinese medicine preparations have been applied to the clinical practice of IPF, and their effectiveness and safety are also widely concerned. Traditional Chinese medicine has obvious clinical effect and less adverse effect on IPF, and has significant effect on helping patients to improve clinical symptoms and quality of life. In recent years, a number of RCT have been completed to verify the total effective rate, lung function and curative effect of traditional Chinese medicine in treating IPF, and a number of systematic reviews (SRs) based on traditional Chinese medicine in treating IPF have been published. However, the relevant SR was published in different years, with various kinds of traditional Chinese medicines included, different outcome indicators and no unified conclusion. At present, there is still a lack of reevaluation of systematic evaluation of traditional Chinese medicine treatment IPF. Therefore, a systematic re-evaluation of the systematic evaluation of traditional Chinese medicine in the treatment of pulmonary fibrosis may help to understand the effective treatment scheme of traditional Chinese medicine in the treatment of pulmonary fibrosis and provide more reliable evidence for the first-line clinicians to treat novel coronavirus.

## 2 Objective

Based on the fact that novel coronavirus has pulmonary interstitial fibrosis in part of the pathological changes of lung, and the effect of traditional Chinese medicine on novel coronavirus is obvious at present, this study mainly discusses the systematic evaluation of traditional Chinese medicine on pulmonary fibrosis for re-evaluation, and finally analyzes the clinical total effective rate, lung function, curative effect of traditional Chinese medicine symptoms, blood gas analysis and other outcome indicators.

## 3 Methods

### 3.1 Study registration

This protocol was recorded in the International Platform of Registered Systematic Review and Meta-analysis Protocols (INPLASY), registration number INPLASY202060029. And if there are any changes, we will describe it in our full review.

### 3.2 Search methods for identification of studies

We will search 3 foreign electronic databases (Cochrane Library, Embase, PubMed) and 4 Chinese electronic databases (China National Knowledge Infrastructure [CNKI], WangFang Database, Chinese Biomedical Literature Database [CBM], and Chinese Scientific Journal Database [VIP]) to collect potential SRs) from their inceptions to February 2020. The language of publication is limited to Chinese or English. The following search terms will be used: Chines medicine, traditional Chinese medicine, Pulmonary Fibrosis, meta- analysis, meta-analysis, meta analyses, systematic review, SRs, etc. A draft search strategy using PubMed, one of the planned electronic databases to be searched, is presented in Table 1.

**Table 1:**
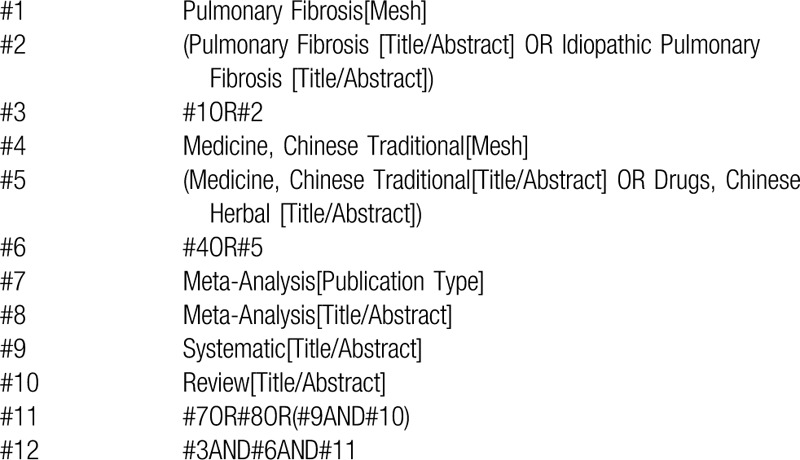
Search strategy (PubMed).

### 3.3 Studies selection

**Figure 1:**
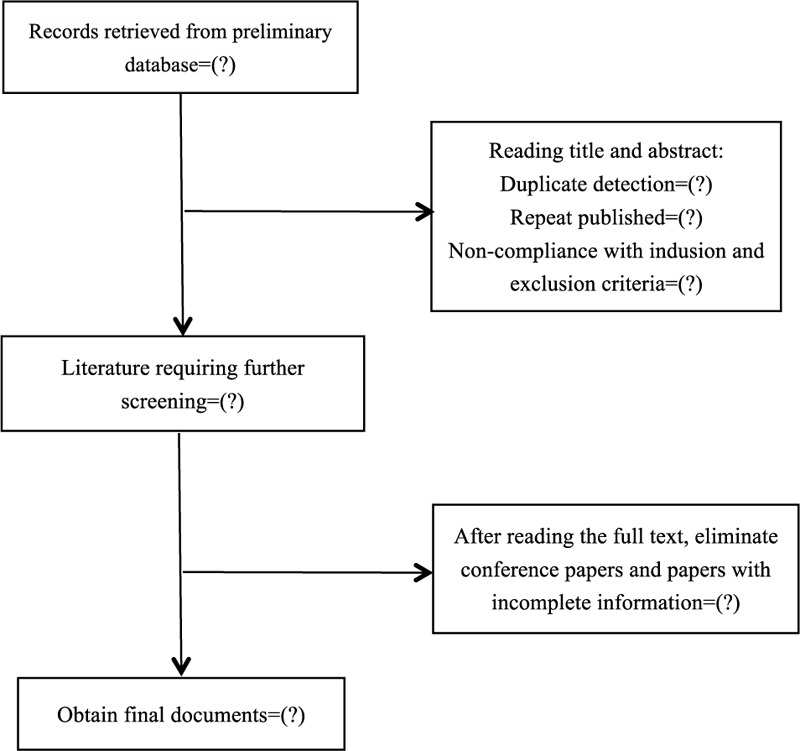
Flowchart of literature selection.

The bibliographies yielded by the literature search will be imported into Endnote for management. Two reviewers (LLJ and CH) will independently read the literature titles, abstracts, and full texts, in sequence, to identify eligible SRs. Any differences will be resolved through discussion to reach a consensus or by using a third author (ZJH) to adjudicate. The planned selection process is shown in a flow chart (Fig. 1)

### 3.4 Inclusion and exclusion criteria

Population, Intervention, Comparison, Outcome and Study (PICOS) strategy was employed.

### 3.5 Types of study

Systematic review/meta-analysis based on randomized controlled trials (RCT).

### 3.6 Type of participants

According to the diagnostic criteria of pulmonary fibrosis, the patient's sex, age, race, onset time and source of cases are not limited.

### 3.7 Type of interventions

Traditional Chinese medicine preparation (such as traditional Chinese medicine decoction, Chinese patent medicine, traditional Chinese medicine monomer, etc.) or traditional Chinese medicine combined with western medicine is used for routine treatment.

### 3.8 Type of control

Use conventional western medicine, placebo or blank control therapy.

### 3.9 Types of outcome measurements

#### 3.9.1 Primary outcomes

Clinical total effective rate, curative effect of TCM symptoms, pulmonary function and blood gas analysis.

#### 3.9.2 Secondary outcomes

6-minute walking test, quality of life, adverse reactions.

#### 3.9.3 Exclusion criteria

① Repeated publication of documents; ② conference papers and comments, etc.; ③ documents whose data cannot be extracted; ④ Intervention measures are other TCM therapies, such as acupuncture, massage, etc. ⑤ Systematic evaluation of other basic diseases with pulmonary fibrosis; ⑥ Non-Chinese and English Literature.

### 3.10 Data extraction

Two reviewers (YWN and XXM) will independently extract the following data: authors’ name, publication year, country, language, sample size, participants, intervention(s), comparison(s), outcome(s), and some relevant characteristics from the full-text. If the data reported is insufficient or missing, WJ will attempt to contact the author for further information to supplement the missing data. In case of any divergence, we will resolve it through discussion and decision by both parties or by consensus with the third reviewer (KYX).

### 3.11 Evaluation of the methodological quality of the included studies

#### 3.11.1 Assessment of multiple systematic reviews-2 measurement tool

This study used AMSTAR2 measurement tool to evaluate the methodological quality of the incorporated system evaluation.^[18]^ multiple system evaluation (AMSTAR) is a reliable methodological quality evaluation tool.^[19]^ during the use of AMSTAR measurement tool, researchers pointed out that the items are difficult to understand and the evaluation options are inappropriate, thus affecting the accuracy of the evaluation results.^[20,21]^ AMSTAR-2 is an update of AMSTAR, which can be used to appraise SRs of both randomized and non-randomized controlled trials.^[22]^ At present, there are 40 quality evaluation tools,^[23]^ among which AMSTAR tool is widely used and is considered to have good reliability, structural validity and practicability.^[24]^ AMSTAR-2 includes 16 items, with each of the 16 criteria given a rating of “yes” (definitely done), “no” (definitely not done), “can’t report” (unclear if completed), or “not applicable” based on information provided by the SRs on which reviewers put an evaluation when the criterion is met.

#### 3.11.2 Preferred reporting items for systematic reviews and meta-analyses (PRISMA) item

In order to reflect the integrity and transparency of this study, Two authors (LLJ and LLY) of the overview will independently evaluate the reporting quality in each review included to assess whether they met the criteria specified in the PRISMA.^[25]^ In case of any difference, it will be settled through discussion between them and arbitrated by a third general author (CX) if necessary.

#### 3.11.3 Risk of bias in systematic review tool

In order to better evaluate the systematic evaluation included in this research, ROBIS is included in this research to evaluate the methodological quality. The development of ROBIS tools is divided into four stages,^[26]^ and the whole process is scientific, rigorous and transparent. ROBIS tool is mainly used to evaluate the bias risk of systematic evaluation. It is not only used to evaluate the bias risk in the production process and result interpretation process of various systematic evaluations including intervention, diagnosis, etiology and prognosis, but also used to evaluate the correlation between systematic evaluation problems and practical problems to be solved by users.^[27]^

#### 3.11.4 Evaluation of the evidence quality of the included studies

The quality of evidence of the included SRs was assessed by the grading of recommendations assessment, development and evaluation (GRADE) approach.^[28]^ The overall quality of evidence was judged as “high,” “moderate,” “low,” or “very low.” Factors leading to RCT degradation include limitations of research, inconsistency of research results, uncertainty as to whether it is direct evidence (indirect or indirect), insufficient accuracy or wide confidence interval (imprecise), and publication bias.

### 3.12 Limitations

Limitations of this study: ① Due to language limitations, this study only included systematic evaluation in both Chinese and English, and did not search for relevant gray literatures, which may lead to the risk of missed detection; ② The number of documents included is limited, and there may be low quality of documents, which may affect the accuracy of results. ③ It is possible that the appraisers are subjective in the evaluation of the most moderate quality, which leads to bias and thus affects the final evaluation results.

## 4 Discussion

The purpose of this study is to reevaluate the existing systematic evaluation of traditional Chinese medicine in the treatment of pulmonary fibrosis. In recent years, a number of RCT have been completed to verify the total effective rate of traditional Chinese medicine in the treatment of IPF, lung function, curative effect of traditional Chinese medicine symptoms, etc., and a number of SR based on traditional Chinese medicine in the treatment of IPF have been published. However, the relevant SR was published in different years, with various kinds of traditional Chinese medicines included, different outcome indicators and no unified conclusion. At present, there is still a lack of reevaluation of systematic evaluation of traditional Chinese medicine treatment IPF. At present, during the period of novel coronavirus, novel coronavirus may lead to the formation of pulmonary interstitial fibrosis, and clinical medication shows that Chinese medicine has obvious effect in treating novel coronavirus. Therefore, this article evaluates the methodological quality and evidence quality of the published SR of Chinese medicine in treating IPF, and provides reference for the prospect and future research of Chinese medicine in treating IPF during the period of novel coronavirus treatment.

## Author contributions

**Conceptualization:** Li Juan Li.

**Data curation:** Xuan Chen, Wen Na Yang.

**Formal analysis:** Li Ying Lu, Xiang Mei Xu.

**Investigation:** Yi Xuan Kong, Jie Wang.

**Methodology:** Li Juan Li, Xuan Chen, Xiang Mei Xu.

**Software:** Yi Xuan Kong, Li Ying Lu.

**Writing – original draft:** Li Juan Li, Xuan Chen, Wen Na Yang, Xiang Mei Xu, Li Ying Lu, Yi Xuan Kong, Jie Wang.

**Writing – review & editing:** Li Juan Li, Xuan Chen, Wen Na Yang, Xiang Mei Xu.
